# Supplementary material for: Methodological quality of COVID-19 clinical research
Source: Nat Commun. 2021 Feb 11;12:943. doi: 10.1038/s41467-021-21220-5 (PMC7878793; doi:10.1038/s41467-021-21220-5)
Supplement: Supplementary file 1 — Supplementary Information [file 41467_2021_21220_MOESM1_ESM.pdf]

Supplementary Information

Methodological Quality of COVID-19 Clinical Research

Jung and Di Santo et al.

University of Ottawa Heart Institute, Ottawa, Ontario, Canada

CONTENTS

Supplementary Tables 1-3

Supplementary Figures 1-2

Supplementary Table 1. Ovid MEDLINE(R) Search Strategy		
#	Searches	Results
1	exp coronavirus infections/	12186
2	coronavirus/ or exp betacoronavirus/	10086
3	(19nCoV or 2019-nCoV or 2019nCoV or corona virus 2 or coronavirus* 2 or corona* 2019 or COV 2 or COVID19* or COVID-19 or COVID or covid-19 or HCoV-19 or ncov* or nCov-2019 or novel corona* or SARS-COV-2 or SARSCOV-2 or SARSCOV2 or severe acute respiratory syndrome?).mp.	18928
4	((new or novel or "19" or "2019" or Wuhan or Hubei or China or Chinese) adj3 (betacoronavirus* or CoV or HCoV)).ti,ab,kf,ot.	956
5	(coronavirus* or corona virus* or covid).ti,ab,kf,ot.	23017
6	((Wuhan or Hubei) adj5 pneumonia).ti,ab,kf,ot.	133
7	or/1-6	30869
8	limit 7 to yr="2019 -Current"	13814
9	limit 8 to english language	12967
10	exp animals/	23161365
11	exp animal experimentation/ or exp animal experiment/	9378
12	exp models animal/	563307
13	nonhuman/	0
14	exp vertebrate/ or exp vertebrates/	22505010
15	or/10-14	23163305
16	exp humans/	18463173
17	exp human experimentation/ or exp human experiment/	12438
18	16 or 17	18463827
19	15 not 18	4700102
20	9 not 19	12685
21	(comment or newspaper article or editorial or letter or note).pt.	1857701
22	20 not 21	8796

Supplementary Table 2. Embase Classic+Embase Search Strategy		
#	Searches	Results
1	exp Coronavirus infection/	13012
2	coronavirinae/ or exp betacoronavirus/	10698
3	(19nCoV or 2019-nCoV or 2019nCoV or corona virus 2 or coronavirus* 2 or corona* 2019 or COV 2 or COVID19* or COVID-19 or COVID or covid-19 or HCoV-19 or ncov* or nCov-2019 or novel corona* or SARS-COV-2 or SARSCOV-2 or SARSCOV2 or severe acute respiratory syndrome?).mp.	19343
4	((new or novel or "19" or "2019" or Wuhan or Hubei or China or Chinese) adj3 (betacoronavirus* or CoV or HCoV)).ti,ab.	843
5	(coronavirus* or corona virus* or covid).ti,ab.	20863
6	((Wuhan or Hubei) adj5 pneumonia).ti,ab.	124
7	or/1-6	30832
8	limit 7 to yr="2019 -Current"	11318
9	limit 8 to english language	10708
10	exp animals/	27649640
11	exp animal experimentation/ or exp animal experiment/	2548806
12	exp models animal/	1373100
13	nonhuman/	6187094
14	exp vertebrate/ or exp vertebrates/	26840780
15	or/10-14	29497971
16	exp humans/	22226816
17	exp human experimentation/ or exp human experiment/	496553
18	16 or 17	22228574
19	15 not 18	7270503
20	9 not 19	9455
21	(comment or newspaper article or editorial or letter or note).pt.	2561942
22	20 not 21	5756
23	(conference abstract or conference review).pt.	3785494
24	22 not 23	5661

Supplementary Table 3. Ovid Cochrane Central Register of Controlled Trials Search Strategy		
#	Searches	Results
1	exp coronavirus infections/	157
2	coronavirus/ or exp betacoronavirus/	2
3	(19nCoV or 2019-nCoV or 2019nCoV or corona virus 2 or coronavirus* 2 or corona* 2019 or COV 2 or COVID19* or COVID-19 or COVID or covid-19 or HCoV-19 or ncov* or nCov-2019 or novel corona* or SARS-COV-2 or SARSCOV-2 or SARSCOV2 or severe acute respiratory syndrome?).mp.	395
4	((new or novel or "19" or "2019" or Wuhan or Hubei or China or Chinese) adj3 (betacoronavirus* or CoV or HCoV)).ti,ab.	35
5	(coronavirus* or corona virus* or covid).ti,ab.	384
6	((Wuhan or Hubei) adj5 pneumonia).ti,ab.	14
7	or/1-6	473
8	limit 7 to yr="2019 -Current"	330

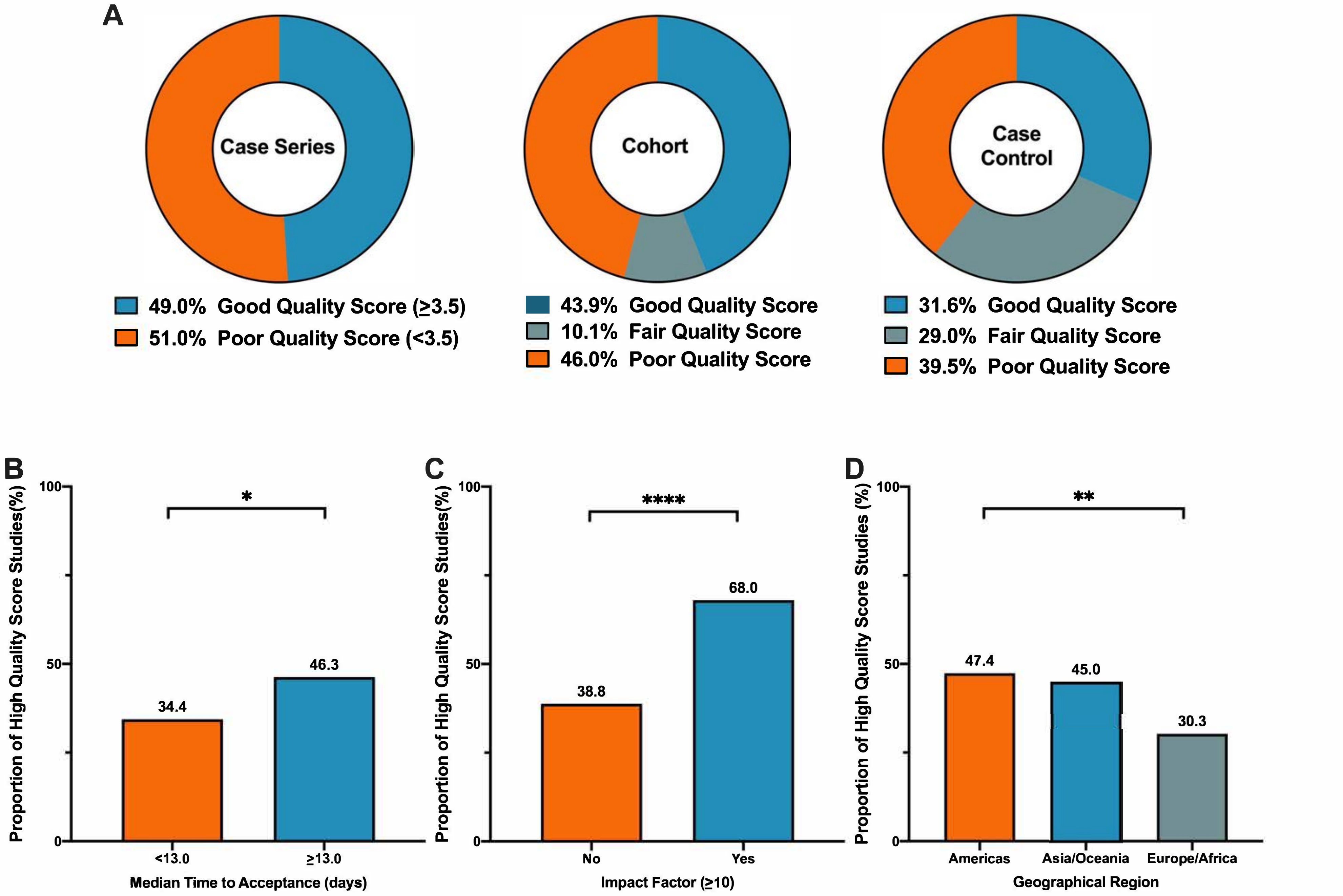

Supplementary Figure 1. COVID-19 clinical literature quality assessment. (A) Overall good quality/low-bias studies was observed for 49.0% of case series, 43.9% of cohort, and 31.6% of case-control studies. (B) Median time to acceptance <13.0 days was associated with lower proportion of high study quality score (34.4% vs. 46.3%, p=0.01). (C) Lower journal impact factor (<10) was associated with lower study quality score (38.8% vs 68.0%, p<0.0001). (D) Studies originating in Americas or Asia/Oceania had was associated with a higher quality score compared to that of Europe/Africa (47.4% vs. 45.0% vs. 30.3% for Americas, Asia/ Oceania, Europe, respectively, p=0.01). In Panel A, orange represents poor, teal represents fair, and blue represents good quality score. In Panels B-C, orange is low and blue is high time to acceptance or impact factor. In Panel D, orange is Americas, blue is Asia/Oceania, and teal is Europe. Chi-Squares Test was conducted to evaluate differences in study quality by median time to acceptance, impact factor, and geographic region. P<0.05 was considered statistically significant.

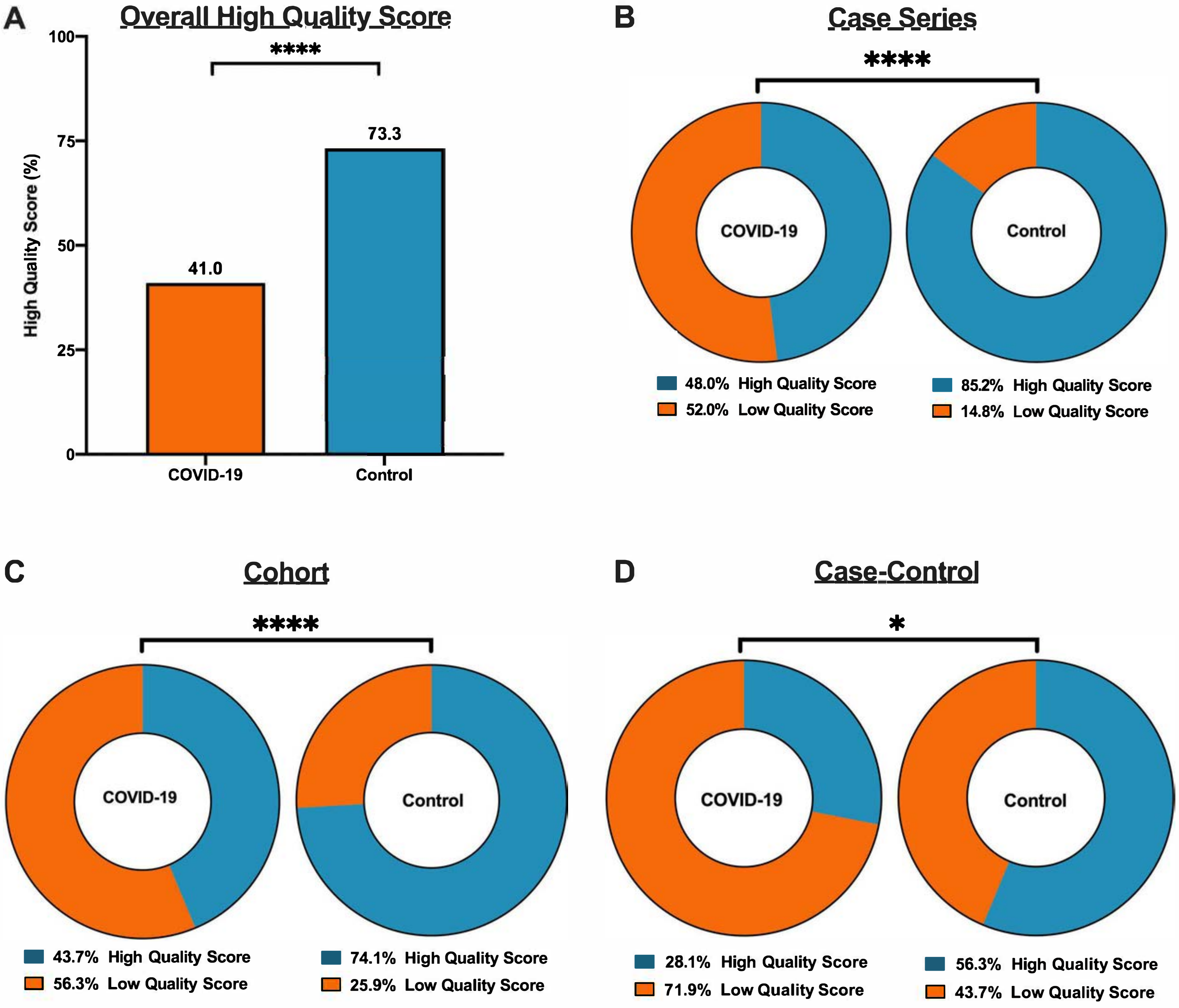

Supplementary Figure 2. Differences in methodological quality in COVID-19 versus historical control article. (A) Lower methodological quality score was associated with COVID-19 articles compared to control articles (220 (41.0%) vs. 392 (73.3%), p<0.0001). (B) Lower quality case series studies score was associated with COVID-19 articles compared to control articles (133 (48.0%) vs. 236 (85.2%), p<0.0001). (C) Lower quality NOS cohort studies score was associated with COVID-19 articles compared to control articles (76 (43.7%) vs. 129 (74.1 %), p<0.0001). (D) Lower quality NOS case-control studies score was associated iwth COVID-19 articles compared to control articles (9 (28.1 %) vs. 18 (56.3%), p=0.02). In Panel A, orange is COVID-19 articles and blue is control articles. In Panels B-D, orange is low and blue is high quality score. Differences in high study quality was evaluated by Chi-Squares Test. P<0.05 was considered statistically significant.
