# Supplementary material for: Methodological quality of COVID-19 clinical research
Source: Nat Commun. 2021 Feb 11;12:943. doi: 10.1038/s41467-021-21220-5 (PMC7878793; doi:10.1038/s41467-021-21220-5)
Supplement: Supplementary file 3 — Description of Additional Supplementary Files [file 41467_2021_21220_MOESM3_ESM.docx]

**Description of Additional Supplementary Files**

**File Name**: Supplementary Data 1
**Description:** Identified COVID-19 clinical manuscripts.

**File Name:** Supplementary Data 2
**Description:** Identified and matched COVID-19 and historical control manuscripts.
