# Supplementary material for: Methodological quality of COVID-19 clinical research
Source: Nat Commun. 2021 Feb 11;12:943. doi: 10.1038/s41467-021-21220-5 (PMC7878793; doi:10.1038/s41467-021-21220-5)
Supplement: Supplementary file 6 — Reporting Summary [file 41467_2021_21220_MOESM6_ESM.pdf]

nature research

Corresponding author(s): Benjamin Hibbert

Last updated by author(s): Dec 20, 2020

Reporting Summary

Nature Research wishes to improve the reproducibility of the work that we publish. This form provides structure for consistency and transparency in reporting. For further information on Nature Research policies, see our Editorial Policies and the Editorial Policy Checklist.

Statistics

For all statistical analyses, confirm that the following items are present in the figure legend, table legend, main text, or Methods section.

n/a	Confirmed	
☐	☒	The exact sample size (*n*) for each experimental group/condition, given as a discrete number and unit of measurement
☐	☒	A statement on whether measurements were taken from distinct samples or whether the same sample was measured repeatedly
☐	☒	The statistical test(s) used AND whether they are one- or two-sided<br>*Only common tests should be described solely by name; describe more complex techniques in the Methods section.*
☐	☒	A description of all covariates tested
☐	☒	A description of any assumptions or corrections, such as tests of normality and adjustment for multiple comparisons
☐	☒	A full description of the statistical parameters including central tendency (e.g. means) or other basic estimates (e.g. regression coefficient) AND variation (e.g. standard deviation) or associated estimates of uncertainty (e.g. confidence intervals)
☐	☒	For null hypothesis testing, the test statistic (e.g. *F*, *t*, *r*) with confidence intervals, effect sizes, degrees of freedom and *P* value noted<br>*Give P values as exact values whenever suitable.*
☐	☒	For Bayesian analysis, information on the choice of priors and Markov chain Monte Carlo settings
☐	☒	For hierarchical and complex designs, identification of the appropriate level for tests and full reporting of outcomes
☐	☒	Estimates of effect sizes (e.g. Cohen's *d*, Pearson's *r*), indicating how they were calculated

Our web collection on statistics for biologists contains articles on many of the points above.

Software and code

Policy information about availability of computer code

Data collection	Data search strategy was designed in MEDLINE and additional searches were conducted in Embase and Cochrane Central Register of Controlled Trials using keywords related to COVID-19. Manuscripts were screened on Covidence and data collection was conducted on Microsoft Excel by two independent reviewers. A historical control group was generated by identifying reports of the same study design of the same journal as that of the COVID-19 manuscript matched in a 1:1 fashion.<br><br>Following the completion of full-text extraction of COVID-19 articles, we obtained a historical control group by identifying reports matched in a 1:1 fashion. From the eligible COVID-19 article, historical controls were identified by searching the same journal in a systematic fashion by matching the same study design ("case series", "cohort", "case control", or "diagnostic") starting in the journal edition 12 months prior to the COVID-19 article publication on the publisher website (ie. COVID-19 article published on April 2020, going backwards to April 2019) and proceeding forward (or backward if a specific article type was not identified) in a temporal fashion until the first matched study was identified following abstract screening by two independent reviewers. If no comparison article was found by either reviewers, the corresponding COVID-19 article was excluded from the comparison analysis. Following the identification of the historical control, data extraction and quality assessment was conducted on the identified articles independently using the standardized case report forms by two reviewers and conflicts resolved by consensus as previously described.<br><br>The primary outcome of interest was to evaluate the methodological quality of COVID-19 clinical literature and control articles by study design using the Newcastle-Ottawa Scale (NOS) for case-control and cohort studies, QUADAS-2 tool for diagnostic studies, Cochrane Risk of Bias for RCTs, and a score derived by Murad et al. for case series studies.

Data analysis	Finalized dataset was collected on Microsoft Excel v16.44. All statistical analyses were performed using SAS v9.4 (SAS Institute, Inc., Cary, NC, USA). Statistical significance was defined as P < 0.05. All figures were generated using GraphPad Prism v8 (GraphPad Software, La Jolla, CA, USA).

For manuscripts utilizing custom algorithms or software that are central to the research but not yet described in published literature, software must be made available to editors and reviewers. We strongly encourage code deposition in a community repository (e.g. GitHub). See the Nature Research guidelines for submitting code & software for further information.

Data

Policy information about availability of data

All manuscripts must include a data availability statement. This statement should provide the following information, where applicable:
- Accession codes, unique identifiers, or web links for publicly available datasets
- A list of figures that have associated raw data
- A description of any restrictions on data availability

The authors declare that all data supporting the findings of the study are available within the paper upon publication. The data has been attached as a supplemental appendix. The original search was conducted on MEDLINE, Embase, and Cochrane Central Register of Controlled Trials and matched articles were identified using the methods described in "data collection".

Field-specific reporting

Please select the one below that is the best fit for your research. If you are not sure, read the appropriate sections before making your selection.

[x] Life sciences [ ] Behavioural & social sciences [ ] Ecological, evolutionary & environmental sciences

For a reference copy of the document with all sections, see nature.com/documents/nr-reporting-summary-flat.pdf

Life sciences study design

All studies must disclose on these points even when the disclosure is negative.

Sample size	A systematic review was conducted on May 14, 2020 to identify COVID-19 clinical manuscripts (n=686) by searching MEDLINE, Embase, and Cochrane Central Register of Controlled Trials. These registries are the standard registries used to systematically evaluate the literature aas per the Cochrane Handbook. We then performed a 1:1 matched cohort study identifying a historical article from the same journal in the previous year in order to evaluate differences in methodological quality (n=539 for both COVID-19 and control articles for a total of 1078 articles).
Data exclusions	The search strategy was published a priori on PROSPERO (CRD42020187318). We designed the inclusion and exclusion criteria in order to identify original research or brief/communication articles pertaining COVID-19 while excluding reviews and editorial articles. As such, we excluded studies which were exploratory or pre-clinical in nature (ie. in vitro or animal studies), case reports, case series <5 patients, studies published in a language other than English, reviews, methods or protocols, and other coronavirus variants such as the Middle East Respiratory Syndrome.
Replication	No replication has yet been conducted as it is a systematic review and matched cohort analysis which has been reviewed by two independent reviewers.
Randomization	Randomization is not applicable to the systematic review and matched cohort analysis.
Blinding	Blinding is not applicable to the systematic review and matched cohort analysis as the manuscripts were analyzed by two independent reviewers unaware of their scores prior to adjudication.

Reporting for specific materials, systems and methods

We require information from authors about some types of materials, experimental systems and methods used in many studies. Here, indicate whether each material, system or method listed is relevant to your study. If you are not sure if a list item applies to your research, read the appropriate section before selecting a response.

Materials & experimental systems

n/a	Involved in the study	
[x]	[ ]	Antibodies
[x]	[ ]	Eukaryotic cell lines
[x]	[ ]	Palaeontology and archaeology
[x]	[ ]	Animals and other organisms
[x]	[ ]	Human research participants
[ ]	[x]	Clinical data
[x]	[ ]	Dual use research of concern

Methods

n/a	Involved in the study	
[x]	[ ]	ChIP-seq
[x]	[ ]	Flow cytometry
[x]	[ ]	MRI-based neuroimaging

Clinical data

Policy information about clinical studies

All manuscripts should comply with the ICMJE guidelines for publication of clinical research and a completed CONSORT checklist must be included with all submissions.

Clinical trial registration	N/A
Study protocol	Study protocol may be found at PROSPERO: CRD42020187318
Data collection	Manuscripts were first screened by two independent reviewers using Covidence and articles that were selected for full review were independently reviewed for quality assessment. Methodological quality was assessed using established quality scores (ie. Newcastle-Ottawa Scale for case-control and cohort studies, QUADAS-2 for diagnostic studies, Cochrane Risk of Bias for randomized controlled trials, and a score derived by Murad et al. for case-series).
Outcomes	All primary and secondary outcomes were determined a priori and registered on PROSPERO (CRD42020187318) prior to data extraction to evaluate methodological rigor of COVID-19 articles against a historical control. Primary outcome of interest was to evaluate the quality of COVID-19 by study type using Newcastle-Ottawa Scale (NOS) for case-control and cohort studies, QUADAS-2 tool for diagnostic studies, Cochrane Risk of Bias for RCTs, and a score derived by Murad et al. for case series. Pre-specified secondary outcomes were comparison of quality scores by: i) median time to acceptance, ii) impact factor, iii) geographical region, and iv) historical comparator. Time to acceptance was defined as the time between submission to acceptance which captures peer review and editorial decisions.<br><br>Comparison of COVID-19 article to historical control was done by evaluating the differences in quality scores by Kruskal-Wallis Test. Furthermore, Good quality of NOS was defined as 3+ on selection and 1+ on comparability and 2+ on outcome/exposure domains. High quality case series was defined as a score ≥3.5. Geographical region was stratified post-hoc on a continent basis into Asia/Oceania, Europe/Africa, and Americas (North and South America).
